# 

**Published:** 1895-12

**Authors:** 


					﻿THE
Southern Medical Record.
A Monthly Journal of Medicine and Surgery.
Vol. XXV. ATLANTA, GA.. DECEMBER, 1895.	No. l2>
BERN ABD WOLFF, M. D., Editor.
LOUIS H. JONES, M. D., Business Manages.
Associate Editors :
A W. GBIGGS, M. D.	WILLIS F. WESTMOBELAND, M. D.
j. McFadden gaston, μ. d.
Entered at the Post-office at Atlanta, <;a., as Second-Class Matter.
The Foote & Davies Co., Atlanta, Ga.
				

